# Restoration of mRNA Expression of Solute Carrier Proteins in Liver of Diet-Induced Obese Mice by Metformin

**DOI:** 10.3389/fendo.2021.720784

**Published:** 2021-09-30

**Authors:** Jiamei Le, Yi Fu, Qiuqin Han, Xindong Wei, Houlin Ji, Yifan Chen, Qiuying Wang, Peixian Pi, Jilei Li, Xinjie Lin, Xiaoying Zhang, Yong Zhang, Jianping Ye

**Affiliations:** ^1^ Shanghai University of Medicine & Health Sciences Affiliated Zhoupu Hospital, Shanghai, China; ^2^ Shanghai Key Laboratory of Molecular Imaging, Collaborative Innovation Center for Biomedicine, Shanghai University of Medicine & Health Sciences, Shanghai, China; ^3^ Department of Surgical Oncology, Nanjing University of Chinese Medicin Affiliated 81st Hospital, Nanjing, China; ^4^ Graduate School, Shanghai University of Traditional Chinese Medicine, Shanghai, China; ^5^ Metabolic Disease Research Center, Zhengzhou Central Hospital Affiliated to Zhengzhou University, Zhengzhou, China; ^6^ Center for Advanced Medicine, College of Medicine, Zhengzhou University, Zhengzhou, China; ^7^ Shanghai Diabetes Institute, Shanghai Jiaotong University Affiliated Sixth People’s Hospital, Shanghai, China

**Keywords:** metformin, transcriptome, solute carrier transporter, insulin sensitivity, obesity

## Abstract

Metformin (MET), the most common medicine for type 2 diabetes (T2DM), improves insulin sensitivity by targeting the liver, intestine and other organs. Its impact on expression of the solute carrier (*Slc*) transporter genes have not been reported in the mechanism of insulin sensitization. In this study, we examined *Slc* gene expression in the liver and colon of diet-induced obese (DIO) mice treated with MET by transcriptomic analysis. There were 939 differentially expressed genes (DEGs) in the liver of DIO mice *vs* lean mice, which included 34 *Slc* genes. MET altered 489 DEGs in the liver of DIO mice, in which 23 were *Slc* genes. Expression of 20 MET-responsive *Slc* DEGs was confirmed by qRT-PCR, in which 15 *Slc* genes were altered in DIO mice and their expressions were restored by MET, including *Slc2a10*, *Slc2a13*, *Slc5a9, Slc6a14*, *Slc7a9*, *Slc9a2*, *Slc9a3*, *Slc13a2*, *Slc15a2*, *Slc26a3*, *Slc34a2*, *Slc37a1*, *Slc44a4*, *Slc51b* and *Slc52a3*. While, there were only 97 DEGs in the colon of DIO mice with 5 *Slc* genes, whose expression was not restored by MET. The data suggest that more genes were altered in the liver over the colon by the high fat diet (HFD). There were 20 *Slc* genes with alteration confirmed in the liver of DIO mice and 15 of them were restored by MET, which was associated with improvement of insulin sensitivity and obesity. The restoration may improve the uptake of glucose, amino acids, mannose, fructose, 1,5-anhydro-D-glucitol and bumetanide in hepatocytes of the liver of DIO mice. The study provides new insight into the mechanism of metformin action in insulin sensitization and obesity.

## Introduction

Metformin (MET), a guanidine derivative initially extracted from the plant *Galega officinalis* (French lilac), is the first-line medicine in the treatment of type 2 diabetes (T2DM) (1). In addition, MET activity has been reported in the treatment of many other diseases, such as cancer, obesity, nonalcoholic fatty liver disease (NAFLD) and inflammation (2). In the treatment of T2DM, MET is considered to act in the liver by inhibition of gluconeogenesis through a couple of mechanisms, such as inhibition of mitochondrial redox shuttle (3), suppression of the Complex I of mitochondrial respiratory chain (4), activation of the cellular energy sensor AMP-activated protein kinase (AMPK) (5). Additionally, MET is reported to act in the intestine to regulate bile acids (6), microbiota (7) and GLP-1 secretion (8) in the improvement of insulin sensitivity. However, the relative importance of liver and intestine remains to be established in the mechanism of insulin sensitization by MET.

Solute carrier (*Slc*) proteins are a group of transmembrane transporters that mediate solute influx and efflux across the plasma and intracellular membranes. The members of *Slc*5, *Slc*13, *Slc*16, *Slc*25, and *Slc*30 families, which are studied in the liver, intestine, pancreas, skeletal muscle, adrenal glands, adipose tissue, etc., have been linked to the metabolic disease, such as obesity, NAFLD and T2DM in human and mouse studies (9). It was reported that *Slc*22A1, *Slc*22A2, *Slc*22A3, *Slc*47A1 played an important role in the kidney, fat and liver for bioavailability, clearance, and pharmacological action of metformin in T2DM (10–12). However, systemic examination of *Slc* genes has not been reported in obesity.

MET inhibits hepatic gluconeogenesis through a direct action in hepatocytes. It is up taken by the organic cation transporters (*Slc*22A1 and *Slc*22A3) and secreted to the bile through an efflux transporter (*Slc*47A1). DNA methylation of these transporter genes (*Slc*22A1, *Slc*22A 3 and *Slc*47A1) are reduced by MET in the human liver, and increased by hyperglycemia and obesity (13). Metformin increases free fatty acid (FFA) uptake under hypoxic conditions, partially through up-regulation of fatty acid transporter *Slc*27A4 gene expression in the rat L6 skeletal muscle cells (14). However, an impact of MET in expression of the *Slc* transporters has not been examined extensively using transcriptome in the liver and colon. In this study, we systematically investigated the MET impact in the liver and colon of DIO mice using transcriptomic analysis, and provided new insight into the mechanism of metformin action in *Slc* mRNA expression.

## Materials and Methods

### Animals and Materials

Male C57BL/6J mice at 8 weeks of age (SPF grade) were obtained from the Shanghai Slac Laboratory Animal Co. Ltd. (Shanghai, China). The mice were kept in the animal facility of Shanghai Jiao Tong University with a controlled temperature (20 ± 2°C), humidity (60 ± 5%) and a 12 h dark/light cycle. The control mice were fed a regular chow diet (NCD mice, n=10, A5002,13.5% Kcal from fat, Shanghai Slac Laboratory Animal Co. Ltd). A total of 20 mice were fed on HFD (# D12492, 60% Kcal from fat, Research diets) for 16 weeks to generate DIO mice as previously described (15). The DIO mice were divided randomly into 2 groups: the HFD control group (HFD mice, n=10) and the MET treated HFD group (HFD+MET mice, n=10). MET was administrated at 100 mg/kg/day through the drinking water. MET (metformin hydrochloride) was purchased from Sigma-Aldrich Co. Ltd. (Shanghai, China). The mice were treated with MET for 8 weeks.

At the end of the treatment, the mice were subjected to tissue collection under anesthesia with intraperitoneal injection of pentobarbital (35 mg/kg). Orbital bleeding was applied in the blood collection. Serum was isolated by centrifugation at 3000 g at 4°C for 10 min and stored at −80°C until the biochemical assays. The mice were sacrificed by cervical dislocation after blood collection. The visceral adipose tissues, livers and colons were collected from the animals and immediately weighed. The samples were flushed with phosphate-buffered saline (PBS, pH7.4) and instantly frozen in liquid nitrogen and then stored at −80°C until subsequent analysis. The animal experiments were conducted in accordance with the protocol approved by the Institutional Animal Care and Use Committee (IACUC) of Shanghai Jiao Tong University. Other chemicals were purchased from Sigma-Aldrich Co. Ltd. (Shanghai, China) unless stated otherwise.

### Histological Analysis

The livers were fixed in 10% phosphate-buffered formalin acetate at 4°C overnight and embedded in paraffin wax. Paraffin sections (5 μm) were cut and mounted on glass slides for hematoxylin and eosin (H&E) staining. Cryosections of the livers were stained by oil red O and counterstained with hematoxylin to visualize the lipid droplets.

### Glucose Tolerance Test and Insulin Tolerance Test

Glucose tolerance test (GTT) and Insulin tolerance test (ITT) were respectively performed in the mice after 16 h fasting with peritoneal injection of glucose (2 g/kg) and 6 h fasting with peritoneal injection of insulin (1 U/kg). Blood glucose was tested in the tail vein blood at 0, 15, 30, 60, and 120 min using a One Touch glucometer (ACCU-CHEK^®^ performa, Roche).

### Insulin Sensitivity and Blood Lipids

The insulin sensitivity index HOMA-IR [=fasting insulin (mU/l) × fasting glucose (mmol/l)/22.5] was calculated according to the fasting insulin and glucose concentration as previously described (15). Blood lipid profile was examined for serum triglyceride, total cholesterol, high-density lipoprotein cholesterol, and low-density lipoprotein cholesterol using an autoanalyzer (Hitachi 7600-020, automatic analyzer).

### RNA Extraction

Total RNA from liver and colon were extracted using Trizol reagent kit (Invitrogen, Carlsbad, CA, USA) according to manual instruction. About 60 mg of tissues were ground into powder by liquid nitrogen in a 2 mL tube, followed by being homogenized for 2 minutes and rested horizontally for 5 minutes. The mix was centrifuged for 5 minutes at 12,000×g at 4°C, then the supernatant was transferred into a new EP tube with 0.3 mL chloroform/isoamyl alcohol (24:1). The mix was shacked vigorously for 15s, and then centrifuged at 12,000×g for 10 minutes at 4°C. After centrifugation, the upper aqueous phase where RNA remained was transferred into a new tube with equal volume of supernatant of isopropyl alcohol, then centrifuged at 13,600 rpm for 20 minutes at 4°C. After deserting the supernatant, the RNA pellet was washed twice with 1 mL 75% ethanol, then the mix was centrifuged at 13,600 rpm for 3 minutes at 4°C to collect residual ethanol, followed by the pellet air dry for 5-10 minutes in the biosafety cabinet. Finally, 25μL~100μL of DEPC-treated water was added to dissolve the RNA. Subsequently, total RNA was qualified and quantified using a Nano Drop and Agilent 2100 bioanalyzer (Thermo Fisher Scientific, MA, USA).

### mRNA Library Construction and Sequencing

Oligo(dT)-attached magnetic beads were used to purified mRNA. Purified mRNA was fragmented into small pieces with fragment buffer at appropriate temperature. Then First-strand cDNA was generated using random hexamer-primed reverse transcription, followed by a second-strand cDNA synthesis. afterwards, A-Tailing Mix and RNA Index Adapters were added by incubating to end repair. The cDNA fragments obtained from previous step were amplified by PCR, and products were purified by Ampure XP Beads, then dissolved in EB solution. The product was validated on the Agilent Technologies 2100 bioanalyzer for quality control. The double stranded PCR products from previous step were heated denatured and circularized by the splint oligo sequence to get the final library. The single strand circle DNA (ssCir DNA) was formatted as the final library. The final library was amplified with phi29 to make DNA nanoball (DNB) which had more than 300 copies of one molecular, DNBs were loaded into the patterned nanoarray and pair end 100 bases reads were generated on BGIseq500 platform (BGI-Shenzhen, China).

### RNA-Seq Data Quality Analysis

In order to ensure data quality for the following analyses, the raw data of 9 samples were firstly filtered with SOAPnuke (v1.5.2) (https://github.com/BGI-flexlab/SOAPnuke) (16). Finally, clean data were obtained by (1) removing reads sequencing adapter, (2) removing reads whose low-quality base ratio (base quality less than or equal to 5) is more than 20%, (3) removing reads whose unknown base (‘N’ base) ratio is more than 5%. All the downstream analyses were based on the clean data with high quality.

### Differentially Expressed Genes Analysis

The differentially expressed genes (DEGs) analysis was performed using the DESeq2 (v1.4.5) (http://www.bioconductor.org/packages/release/bioc/html/DESeq2.html) (17). We identified genes with a fold change (FC) ≥ 2 and Q value ≤ 0.05 in a comparison as significant DEGs.

### Gene Ontology and KEGG Pathway Enrichment Analysis

To take insight to the change of phenotype, Gene Ontology (GO) (http://www.geneontology.org/) and Kyoto Encyclopedia of Genes and Genomes (KEGG) (https://www.kegg.jp/) enrichment analysis of annotated different expressed gene was performed by Phyper (https://en.wikipedia.org/wiki/Hypergeometric_distribution) based on Hypergeometric test. The significant levels of terms and pathways were corrected by Q value with a rigorous threshold (Q value ≤ 0.05), as previously described (18).

### Real Time Quantitative RT-PCR

To confirm the transcriptomic data, MET-responsive *Slc* transporters DEGs were selected and validated through qRT-PCR. Total RNA was extracted using the EastepTM Total RNA Super Extraction Kit (promega, Shanghai, China) according to the manufacturer’s instruction and quantified with a Denovix DS-11 Spectrophotometer (Denovix, Inc., Wilmington DE, USA). cDNA was synthesized from total RNA (1 μg: 20 μl final reaction volume) using ReverTra Ace^®^ qPCR RT Master Mix with gDNA Remover (TOYOBO Bio-Technology, CO., Shanghai, China) in a SimpliAmp Thermal Cycler (Applied Biosystems, Thermo Fisher Scientific, Inc., Waltham, MA, USA). A 20 μl PCR reaction system included 2 μl cDNA, 10 μl TB mixture, 0.4 μl forward primer, 0.4 μl reverse primer, 0.4 μl ROX Reference Dye II and 6.8 μl deionized water. After mixing, the PCR reaction was performed using ABI Prism TM 7500 Real-Time qPCR System (Applied Biosystems; Thermo Fisher Scientific, Inc.). The GAPDH was used as a house gene to normalize the expression level of the test genes, and the relative gene expression level was analyzed using the 2^−ΔΔCT^ method. All samples were analyzed in triplicate. Primers were synthesized by GENEWIZ (Suzhou, China) and were listed in the **Supplementary Table 1**.

### Statistical Analysis

The results are expressed as the mean ± SEM. The tissue weight, serum blood lipid levels, and mRNA expression were analysed using one-way ANOVA followed by Brown-Forsythe and Welch multiple comparisons tests. The body weight, fasting blood glucose, fasting serum insulin, HOMA-IR index, AUC_glucose_ during GTT and ITT were analysed by two-way (repeated measures) ANOVA followed by Holm-Šídák multiple comparisons tests (note: each time was analysed separately). All statistical analyses were performed using GraphPad Prism 9.0 software (La Jolla, CA, USA) with a statistical significance set at *P* < 0.05.

## Results

### Inhibitory Effect of MET on Hepatic Steatosis, Hyperlipidemia, and Obesity

**Figure 1:**
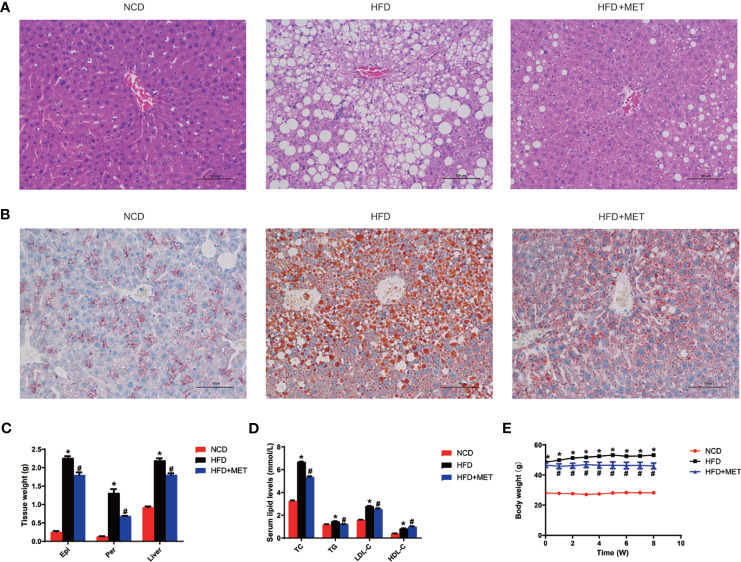
Inhibition of hepatic steatosis, hyperlipidemia, and obesity by MET. **(A)** Liver hematoxylin and eosin (H&E) staining. **(B)** Liver oil red O staining, ×200 (scale bars, 100 μm). **(C)** Tissue weight of perirenal fat, epididymal fat and liver after 8 weeks of treatment. **(D)** Serum lipid levels after 8 weeks of treatment. **(E)** Curve of body weight change from 0 to 8 weeks of treatment. The MET treatment was administrated for 8 weeks in HFD mice after 16 weeks on high-fat diet. Data are presented as the mean ± SEM (n = 10). ^*^ *P* < 0.05 HFD *versus* NCD, ^#^ *P* < 0.05 HFD+MET *versus* HFD.

The DIO mice were generated in C57BL/6 mice with HFD feeding for 16 wks. In the MET-treated DIO mice, the hepatic steatosis was decreased for a reduction in the hepatic intracellular vacuoles observed by H&E staining and oil red O staining of the liver tissue (**Figures 1A, B** **)**. The pathological changes in liver for pale fatty color, the number of hepatic fat vacuoles and hepatomegaly were reversed by MET (**Figures 1A–C**). A reduction in the fat mass (epididymal and perirenal fat) and liver weight were observed in the MET-treated mice (**Figure 1C**). An improvement in hyperlipidemia was observed with parameters including the low-density lipoprotein C, total cholesterol, and total triglyceride (**Figure 1D**). Moreover, the high-density lipoprotein C was increased (**Figure 1D**). A reduction in the body weight gain was observed in the MET-treated group as indicated by the weekly body weight data (**Figure 1E**). These data suggest that the model was successfully established for MET efficacy in the DIO mice.

### Improvement of Insulin Sensitivity by MET

**Figure 2:**
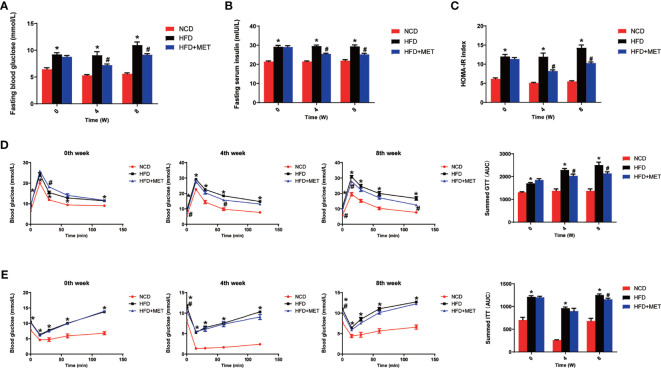
Improvement of insulin sensitivity by MET. **(A)** Fasting blood glucose. **(B)** Fasting serum insulin. **(C)** HOMA-IR. **(D)** GTT at 0, 4, 8 weeks of MET treatment. **(E)** ITT at 0, 4, 8 weeks of MET treatment. GTT and ITT was performed by intraperitoneal injection of glucose and insulin. Data are presented as the mean ± SEM (n = 10). *P < 0.05 HFD *versus* NCD, ^#^ *P* < 0.05 HFD+MET *versus* HFD.

Glucose metabolism was examined in the DIO mice at the 0, 4 and 8 weeks of the MET-treatment. An improvement in insulin sensitivity by MET was supported by the reduction in fasting glucose and fasting insulin (**Figures 2A, B** **)**, which led to a favorite change in the index of HOMA-IR for insulin sensitization (**Figure 2C**). The improvement was extended in other tests including GTT and ITT at 4 and 8 weeks (**Figures 2D, E** **)**. This group of data suggests that the insulin sensitivity was improved by MET in the DIO mice to support the MET efficacy in the DIO model.

### DEGs in liver of DIO mice

**Figure 3:**
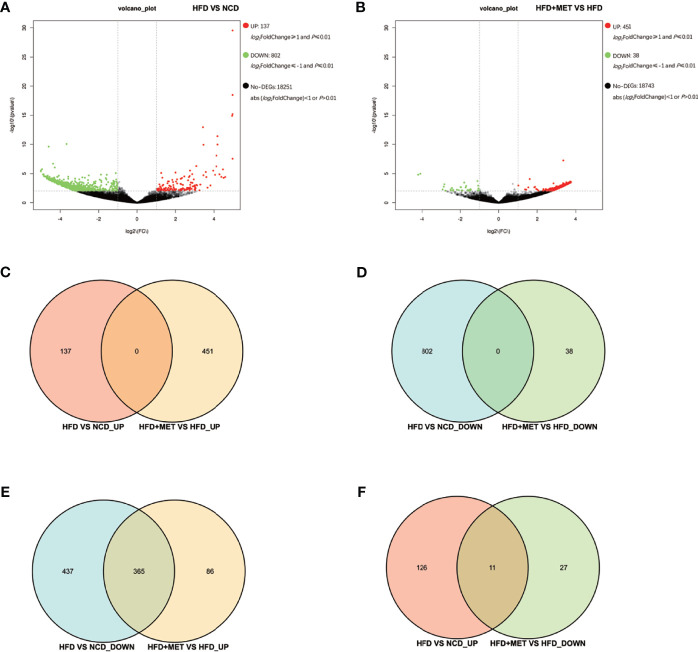
Differentially expressed genes (DEGs) analysis in liver. The volcano plot showed distribution pattern of DEGs in the comparison of HFD *vs* NCD **(A)** and HFD+MET *vs* HFD **(B)**. Venn diagram of upregulated common DEGs **(C)** and downregulated common DEGs **(D)** in two comparisons of HFD *vs* NCD and HFD+MET *vs* HFD. Venn diagram of common DEGs showing opposite expression changes in two comparisons of HFD *vs* NCD and HFD+MET *vs* HFD **(E, F)**. Red color or positive number indicates upregulation, and green color or negative number indicates down-regulation.

The liver gene expression was examined using the RNA-seq technology to investigate the mechanism of MET action. The differentially expressed genes (DEGs) were identified by the gene expression levels determined with fragments per kilobase of exon per million fragments mapped (FPKM) method. The differential expression in all samples was comprehensively analyzed with the criteria of FC≥2 and *P ≤* 0.01. In the liver, 939 DEGs were identified in the DIO mice (HFD *vs* NCD), and 489 DEGs were found in the MET-treated DIO mice (HFD+MET *vs* HFD) (**Figures 3A, B** **)**. In DEGs of DIO mice, 137 were upregulated and 802 downregulated (**Figure 3A**). In the DEGs of MET-treated group, 451 were upregulated and 38 downregulated (**Figure 3B**). Among the HFD-responsive DEGs, there were 34 *Slc* genes with 32 downregulated and 2 upregulated in the DIO mice (HFD *vs* NCD) (**Table 1**), and in the MET-responsive DEGs, there were 23 *Slc* genes upregulated in the MET-treated DIO mice (HFD+MET *vs* HFD) (**Table 2**). Venn diagram analysis of the DEGs revealed that MET did not up-regulate any DEGs that were up in the liver of DIO mice (**Figure 3C**). MET did not down-regulate any DEGs that were down in the DIO mice (**Figure 3D**). However, MET upregulated 365 out of 802 DEGs that were downregulated in the DIO mice (HFD+MET *vs* HFD) (**Figure 3E**). MET downregulated 11 DEGs out of 137 that were upregulated in the liver of DIO mice (HFD+MET *vs* HFD) (**Figure 3F**). mRNA of 20 out of 32 *Slc* genes that were downregulated in the DIO mice were upregulated in the MET-treated DIO mice (**Table 3**). The data suggest that the liver had a dramatic response to HFD with mRNA expression as suggested by DEGs, and MET were able to counter-regulate almost half of them.

**Table 1:** Slc transporters DEGs in liver compared between HFD group and NCD group (\log2FC\≥1 and P<0.01).

Gene ID	Symbol	Description	log_2_FC	P value	Variation trend
20510	Slc1a1	excitatory amino acid transporter 3	-3.58192	0.002005	Down
20514	Slc1a5	neutral amino acid transporter B (0)	-2.55003	0.004563	Down
170441	Slc2a10	solute carrier family 2 (facilitated glucose transporter), member 10	-2.95141	0.002313	Down
239606	Slc2a13	proton myo-inositol cotransporter	-3.63064	0.000488	Down
20537	Slc5a1	unnamed protein product	-3.77914	0.000583	Down
216225	Slc5a8	sodium-coupled monocarboxylate transporter 1	-4.25732	0.000236	Down
230612	Slc5a9	solute carrier family 5 (sodium/glucose cotransporter), member 9, isoform CRA_b, partial	-3.88485	0.001178	Down
15567	Slc6a4	sodium-dependent serotonin transporter	-2.96903	0.003356	Down
102857	Slc6a8	sodium- and chloride-dependent creatine transporter 1 isoform 3; sodium- and chloride-dependent creatine transporter 1 isoform 1; solute carrier family 6 (neurotransmitter transporter, creatine), member 8, isoform CRA_a, partial	-3.50688	0.000971	Down
56774	Slc6a14	sodium- and chloride-dependent neutral and basic amino acid transporter B(0+)	-4.50093	9.50E-05	Down
224022	Slc7a4	cationic amino acid transporter 4 isoform X1	-2.23075	0.000335	Down
30962	Slc7a9	solute carrier family 7 (cationic amino acid transporter, y+ system), member 9, isoform CRA_b, partial	-3.32269	0.006116	Down
226999	Slc9a2	solute carrier family 9 (sodium/hydrogen exchanger), member 2, isoform CRA_a, partial	-4.27668	0.000267	Down
105243	Slc9a3	sodium/hydrogen exchanger 3 precursor	-3.51883	0.001746	Down
20500	Slc13a2	solute carrier family 13 member 2	-4.64711	2.27E-05	Down
790911	Slc13a2os	mCG146185, partial	-3.47566	0.002674	Down
243755	Slc13a4	solute carrier family 13 member 4	2.680519	0.003954	Up
56643	Slc15a1	solute carrier family 15 member 1	-3.90485	0.000646	Down
57738	Slc15a2	solute carrier family 15 member 2 isoform 1; solute carrier family 15 (H+/peptide transporter), member 2, isoform CRA_b	-4.59956	2.39E-10	Down
277898	Slc15a5	solute carrier family 15 member 5	2.394322	0.004192	Up
217316	Slc16a5	monocarboxylate transporter 6	-1.35628	0.001467	Down
24059	Slc21a2	solute carrier organic anion transporter family member 2A1	-1.78776	0.004075	Down
22626	Slc23a3	solute carrier family 23 (nucleobase transporters), member 3, partial	-2.68098	0.006767	Down
229731	Slc25a24	calcium-binding mitochondrial carrier protein SCaMC-1	-3.52426	0.000856	Down
13521	Slc26a2	sulfate transporter	-3.65957	0.001049	Down
13487	Slc26a3	chloride anion exchanger	-4.68552	7.22E-05	Down
20531	Slc34a2	sodium-dependent phosphate transport protein 2B isoform X1	-5.07452	2.78E-06	Down
224674	Slc37a1	glycerol-3-phosphate transporter	-2.94471	0.006447	Down
56857	Slc37a2	solute carrier family 37 (glycerol-3-phosphate transporter), member 2, isoform CRA_a, partial	-4.14538	0.00032	Down
72027	Slc39a4	zinc transporter ZIP4 precursor	-1.60956	0.006416	Down
72002	Slc39a5	solute carrier family 39 (metal ion transporter), member 5, isoform CRA_b, partial	-3.01113	0.001091	Down
70129	Slc44a4	choline transporter-like protein 4	-4.6403	5.94E-05	Down
330962	Slc51b	organic solute transporter subunit beta	-3.8114	0.000104	Down
69698	Slc52a3	solute carrier family 52, riboflavin transporter, member 3 isoform 1 precursor; solute carrier family 52, riboflavin transporter, member 3 isoform 2	-3.69715	0.000307	Down

**Table 2:** Slc transporters DEGs in liver compared between HFD+MET group and HFD group (\log2FC\≥1 and P<0.01).

Gene ID	Symbol	Description	log_2_FC	P value	Variation trend
170441	Slc2a10	solute carrier family 2 (facilitated glucose transporter), member 10	2.976387	0.001975	Up
239606	Slc2a13	proton myo-inositol cotransporter	2.712812	0.006263	Up
20537	Slc5a1	unnamed protein product	3.032958	0.002372	Up
216225	Slc5a8	sodium-coupled monocarboxylate transporter 1	3.207696	0.001732	Up
230612	Slc5a9	solute carrier family 5 (sodium/glucose cotransporter), member 9, isoform CRA_b, partial	2.893464	0.006372	Up
56774	Slc6a14	sodium- and chloride-dependent neutral and basic amino acid transporter B(0+)	3.645746	0.000411	Up
30962	Slc7a9	solute carrier family 7 (cationic amino acid transporter, y+ system), member 9, isoform CRA_b, partial	2.678152	0.009553	Up
226999	Slc9a2	solute carrier family 9 (sodium/hydrogen exchanger), member 2, isoform CRA_a, partial	3.39624	0.00124	Up
105243	Slc9a3	sodium/hydrogen exchanger 3 precursor	2.985228	0.004804	Up
171286	Slc12a8	solute carrier family 12 member 8 isoform 1; solute carrier family 12 member 8 isoform X5	2.600641	0.009193	Up
20500	Slc13a2	solute carrier family 13 member 2	3.493265	0.000674	Up
57738	Slc15a2	solute carrier family 15 member 2 isoform 1; solute carrier family 15 (H+/peptide transporter), member 2, isoform CRA_b	3.353095	5.65E-08	Up
217316	Slc16a5	monocarboxylate transporter 6	1.42968	0.005747	Up
56517	Slc22a21	solute carrier family 22 member 21	2.552414	0.007362	Up
229731	Slc25a24	calcium-binding mitochondrial carrier protein SCaMC-1	3.169503	0.001724	Up
384071	Slc25a34	solute carrier family 25 member 34	1.37105	0.005277	Up
13521	Slc26a2	sulfate transporter	3.081388	0.002561	Up
13487	Slc26a3	chloride anion exchanger	3.250563	0.001134	Up
20531	Slc34a2	sodium-dependent phosphate transport protein 2B isoform X1	2.628209	0.004297	Up
224674	Slc37a1	glycerol-3-phosphate transporter	2.919821	0.002715	Up
70129	Slc44a4	choline transporter-like protein 4	3.65143	0.000433	Up
330962	Slc51b	organic solute transporter subunit beta	3.391635	0.001222	Up
69698	Slc52a3	solute carrier family 52, riboflavin transporter, member 3 isoform 1 precursor; solute carrier family 52, riboflavin transporter, member 3 isoform 2	3.071518	0.00165	Up

**Table 3:** MET-responsive Slc transporters DEGs in liver (\log2FC\≥1 and P<0.01).

Gene ID	Symbol	HFD *VS* NCD			HFD+MET *VS* HFD		
		log_2_FC	P value	Variation trend	log_2_FC	P value	Variation trend
170441	Slc2a10	-2.95141	0.002313	Down	2.976387	0.001975	Up
239606	Slc2a13	-3.63064	0.000488	Down	2.712812	0.006263	Up
20537	Slc5a1	-3.77914	0.000583	Down	3.032958	0.002372	Up
216225	Slc5a8	-4.25732	0.000236	Down	3.207696	0.001732	Up
230612	Slc5a9	-3.88485	0.001178	Down	2.893464	0.006372	Up
56774	Slc6a14	-4.50093	9.50E-05	Down	3.645746	0.000411	Up
30962	Slc7a9	-3.32269	0.006116	Down	2.678152	0.009553	Up
226999	Slc9a2	-4.27668	0.000267	Down	3.39624	0.00124	Up
105243	Slc9a3	-3.51883	0.001746	Down	2.985228	0.004804	Up
20500	Slc13a2	-4.64711	2.27E-05	Down	3.493265	0.000674	Up
57738	Slc15a2	-4.59956	2.39E-10	Down	3.353095	5.65E-08	Up
217316	Slc16a5	-1.35628	0.001467	Down	1.42968	0.005747	Up
229731	Slc25a24	-3.52426	0.000856	Down	3.169503	0.001724	Up
13521	Slc26a2	-3.65957	0.001049	Down	3.081388	0.002561	Up
13487	Slc26a3	-4.68552	7.22E-05	Down	3.250563	0.001134	Up
20531	Slc34a2	-5.07452	2.78E-06	Down	2.628209	0.004297	Up
224674	Slc37a1	-2.94471	0.006447	Down	2.919821	0.002715	Up
70129	Slc44a4	-4.6403	5.94E-05	Down	3.65143	0.000433	Up
330962	Slc51b	-3.8114	0.000104	Down	3.391635	0.001222	Up
69698	Slc52a3	-3.69715	0.000307	Down	3.071518	0.00165	Up

### DEGs in Colon of DIO Mice

**Figure 4:**
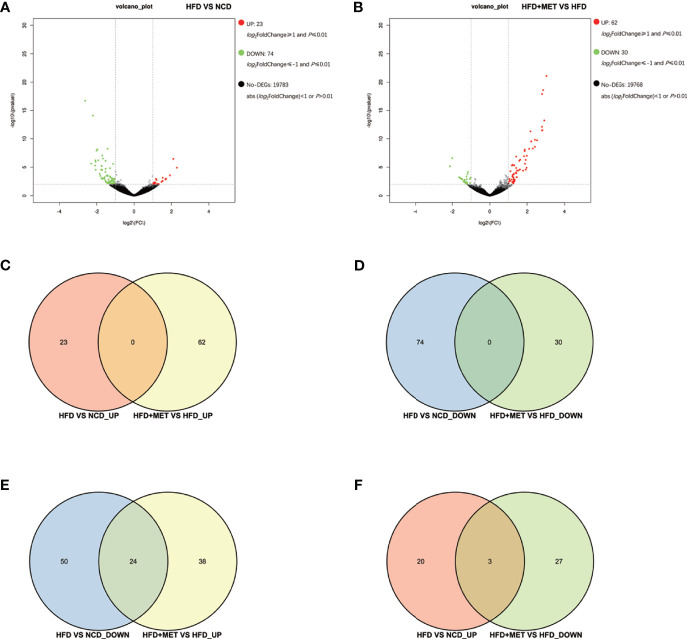
Differentially expressed genes (DEGs) analysis in colon. The volcano plot showed distribution pattern of DEGs in the comparison of HFD *vs* NCD **(A)** and HFD+MET *vs* HFD **(B)**. Venn diagram of upregulated common DEGs **(C)** and downregulated common DEGs **(D)** in two comparisons of HFD *vs* NCD and HFD+MET *vs* HFD. Venn diagram of common DEGs showing opposite expression changes in two comparisons of HFD *vs* NCD and HFD+MET *vs* HFD **(E, F)**. Red color or positive number indicates upregulation, and green color or negative number indicates down-regulation.

The gene expression was examined in the colon tissue in the same mice by RNA-seq to test the impact of MET in intestine. In response to HFD, 97 DEGs were identified in the colon of DIO mice (HFD *vs* NCD) and 92 DEGs were identified in the MET-treated DIO mice (HFD+MET *vs* HFD) with the criteria of FC≥2 and P ≤ 0.01 (**Figures 4A, B** **)**. In the DIO mice, 23 DEGs were upregulated and 74 downregulated (**Figure 4A**). In the MET-treated DIO mice, 62 DEGs were upregulated and 30 downregulated (**Figure 4B**). There were 5 *Slc* genes in the DEGs of DIO mice, and 2 *Slc* genes in the MET-treaded DIO mice, respectively (**Supplementary Table 2**). Venn diagram analysis of the DEGs revealed that HFD-responsive and MET-responsive DEGs did not share the same direction of changes (**Figures 4C, D** **)**. In contrast, MET counter-regulated 24 DEGs that were downregulated in the DIO mice (**Figure 4E**) and 3 DEGs that were upregulated in the DIO mice (**Figure 4F**). Expression of several *Slc* transporters was changed in the DIO, and none of them were restored by MET in the DIO mice (**Supplementary Table 2**). The data suggest that the colon responded to HFD with fewer DGEs, which was 1/8 of those in the liver. MET upregulated 24 downregulated DEGs in the colon of DIO mice and none of them was *Slc* gene.

### GO Enrichment Analysis of MET-Responsive Slc Genes in Liver

**Figure 5:**
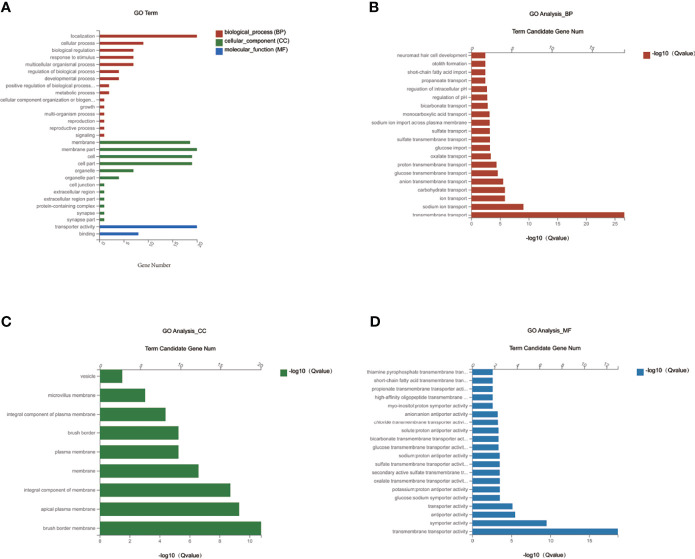
GO enrichment analysis of MET-responsive Slc genes in liver. **(A)** The annotation and classification of GO functional enrichment analysis with the common DEGs of Slc transporters in the liver. The functional enriched classes of the common DEGs of Slc transporters in the liver annotated by biological processes (BP) **(B)**, and cell components (CC) **(C)**, molecular function (MF) **(D)** sub-ontologies of GO enrichment analysis.

To investigate the function of the candidate DEGs of *Slc* transporters, we conducted GO enrichment analyses on the 20 common DEGs of *Slc* transporter genes (**Figure 5**). As shown in **Figure 5A**, the localization, cellular process and biological regulation were the subcategory of highest percentages in the biological processes (BP); the membrane and membrane part were the main significant terms in the cellular component (CC); the transporter activity was the most representative functions in the molecular function (MF). The 20 common DEGs of *Slc* genes in liver were mainly enriched in the BP associated with the transmembrane transport of sodium, ion, carbohydrate, anion transmembrane, glucose transmembrane, proton transmembrane, oxalate, glucose, sulfate, monocarboxylic acid, bicarbonate, propanoate (**Figure 5B**). As for the CC, the *Slc* gene products were mainly enriched in the brush border membrane, apical plasma membrane, integral component of membrane, plasma membrane, integral component of plasma membrane, microvillus membrane and vesicle (**Figure 5C**). The results for MF analysis demonstrated that the *Slc* gene products were mainly related to the functions of transmembrane transporter including symporter, antiporter, glucose: sodium symporter, potassium: porton antiporter, oxalate, secondary active sulfat, sulfate, sodium: proton antiporter, myo-insositol: proton symporter, high-affinity oligopeptide, propionate, short-chain fatty acid, thiamine pyrophosphate (**Figure 5D**). The data suggest that the cross-membrane transporter activities may be decreased by alteration in their mRNA expression in the liver of DIO mice. MET may restore the transporter activities by rescuing the gene expression.

### KEGG Pathway Enrichment Analysis of MET-Responsive Slc Genes in Liver

**Figure 6:**
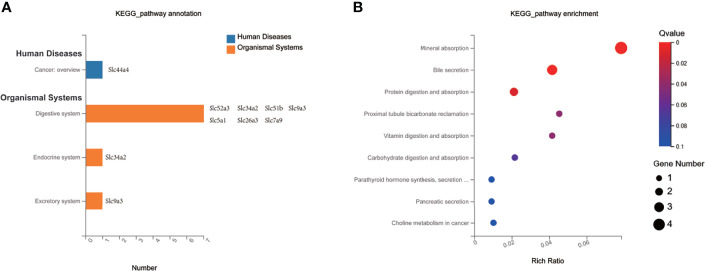
KEGG pathway enrichment analysis of MET-responsive Slc genes in liver. **(A)** KEGG pathway annotation and classification of common Slc transporters DEGs in liver. **(B)** Scatterplot of KEGG enrichment pathways associated with metabolic balance of common Slc transporters DEGs in liver.

To investigate the impact of MET-responsive *Slc* genes in the liver metabolism, we conducted an analysis of the metabolic pathway using the Kyoto Encyclopedia of Genes and Genomes (KEGG). The 20 *Slc* genes were clustered into 2 main categories, including organismal systems and human diseases, which were further divided into 4 subcategories (**Figure 6A**). They were mainly enriched in the mineral absorption, bile secretion, protein digestion and absorption, vitamin digestion and absorption, carbohydrate digestion and absorption, synthesis, secretion and action of parathyroid hormone, pancreatic secretion, which were associated with metabolic balance (**Figure 6B**). The data suggests that HFD may change digestion and absorption of proteins, carbohydrates, minerals, and vitamins in the liver. MET may target these pathways by restoring the expression of *Slc* genes in the improvement of insulin sensitivity.

### Validation of MET-Responsive *Slc* Genes in Liver

**Figure 7:**
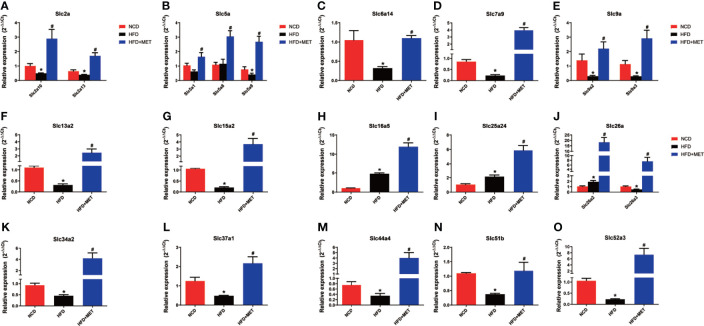
Expression validation of the 20 MET-responsive Slc genes in liver via RT-qPCR. The mRNA levels of the 20 common Slc DEGs were validated by RT-qPCR (2−ΔΔCT method) **(A–O)**. The housekeeping gene GAPDH was used to normalize the relative expression level. Data are presented as the mean ± SEM (n = 3). *P < 0.05 HFD *versus* NCD, ^#^ *P* < 0.05 HFD+MET *versus* HFD.

The 20 MET-responsive *Slc* genes were validated with qRT-PCR assays (**Figure 7**). Among the 20 genes, 15 genes with downregulation in the DIO mice were confirmed in qRT-PCR, which included *Slc2a10*, *Slc2a13*, *Slc5a9, Slc6a14*, *Slc7a9*, *Slc9a2*, *Slc9a3*, *Slc13a2*, *Slc15a2*, *Slc26a3*, *Slc34a2*, *Slc37a1*, *Slc44a4*, *Slc51b* and *Slc52a3* (**Figures 7A–G, J–O**). Their up-regulation by MET was also confirmed in qRT-PCR (**Figures 7A–G** **, J–O**). Three of them with upregulation in the DIO mice (including *Slc16a5*, *Slc25a24* and *Slc26a2*) were confirmed in qRT-PCR and their expression was further upregulated by MET as confirmed in qRT-PCR (**Figures 7H–J**). MET upregulated two *Slc* genes, *Slc5a1* and *Slc5a8*, which were not changed in the untreated DIO mice (**Figure 7B**).

## Discussion

The transmembrane transporters are the gatekeeper proteins, which regulate the import and export of molecules, such as sugars, amino acids, fatty acids, nucleotides, metals, organic anions, inorganic ions, oligopeptides, and drugs (19). They are crucial for cellular homeostasis and their dysfunctions may contribute to the metabolic diseases. In fact, around 10% of all human genes encode the transporter proteins (20). The transporters include the ATP-binding cassette (ABC) transporters, ion channels, and solute carrier proteins (*Slc*s). The *Slc* transporters are the largest group of the transmembrane transporter proteins, which comprise over 60 subfamilies with more than 400 genes. *Slc*s are localized in the membrane surface to mediate passive and secondary active transportation of substrates (20). The *Slc* transporters are highly expressed in the metabolically active organs, such as the liver, kidney, brain, and intestine (9). Their roles in insulin resistance remains largely unknown.

Emerging evidence suggests that *Slc* transporters are closely associated with the metabolic diseases, and are potential candidates of drug targets for insulin resistance, T2DM, hypertension, chronic kidney disease (CKD), gout, asthma, inflammatory bowel disease (IBD), cancer, dementia, and anxiety disorders (9, 21–23). The activities are related to tissue specificity of the *Slc* genes. In our study, the *Slc* genes were examined in the liver and alteration in their expression was associated with hepatic steatosis, hyperlipidemia, obesity and insulin sensitivity in the DIO mice. We identified 20 MET-responsive *Slc* genes *via* transcriptome analysis, whose tissue distribution, physiological functions and pathological roles are shown in the **Supplementary Table 3**. Among them, expression of 15 *Slc* genes that were reduced in the liver of DIO mice was restored by MET. The 15 *Slc* members are *Slc2a10, Slc2a13, Slc5a9, Slc6a14, Slc7a9, Slc9a2, Slc9a3, Slc13a2, Slc15a2, Slc26a3, Slc34a2, Slc37a1, Slc44a4, Slc51b and Slc52a3*. Additionally, mRNA of 3 *Slc* genes (*Slc16a5*, *Slc25a24*, *Slc26a2)* were upregulated in DIO mice and further upregulated in MET-treated DIO mice. Expression of *Slc5a1* and *Slc5a8* were upregulated by MET although they were not altered in the liver of DIO mice. The data suggest that the 15 *Slc* genes may contribute to the mechanism of MET activity in insulin sensitization.

The exact functions of the MET-responsive *Slc* genes are largely unknown in obesity and type 2 diabetes. However, there are reports on six of *Slc* genes (*Slc2a10*, *Slc5A1*, *Slc5a9*, *Slc6A14*, *Slc16A5*, *Slc25A24*) in metabolic diseases including obesity, dyslipidemia, NAFLD, and T2DM. The *Slc2a* gene encodes the glucose transporter (GLUT) that has a family of 13 isoforms (24). *Slc2a10* is also known as *Glut10*, whose expression was reduced in the liver of DIO mice in this study. *Glut10* gene is highly expressed in the pancreas and liver with a function in transportation of glucose (25). However, GLUT10 is found to mediate ascorbic uptake in cells in a recent study. *Glut10* was studied in adipocytes through genetic analysis, in which mutation-induced inactivation of *Glut10* gene impaired adipogenesis and reduced adipose tissue development *via* an ascorbic acid-mediated pathway leading to insulin resistance, suggesting that *Glut10* may mediate ascorbic acid uptake in cells (26). However, the role of *Glut10* (*Slc2a10*) remains unknown in the control of insulin sensitivity in the liver. In our study, the downregulation of *Glut10* gene in the liver of DIO mice was restored by MET-treatment, suggesting that MET may induce ascorbic acid uptake in the liver in the mechanism of insulin sensitization.

The *Slc5a* gene encodes the sodium-dependent glucose transporters (SLGTs) that has a family of 11 isoforms (27). SGLT2 is a well-known member in the kidney for glucose reabsorption from urine, and SGLT2 inhibitors are effective medicines in the treatment of type 2 diabetes for promotion of glucose discharge in urine. *Slc5a1* gene encodes SGLT-1 in the intestine for absorption of glucose (Glc) and galactose (Gal) (28). *Slc5a1* expression is reported to be reduced by the inhibitors of mitochondrial electron transport chain complexes (such as metformin, rotenone and antimycin A) in mouse ileal cultures mouse duodenal organoid derived 2D monolayer cultures (29). In human, *Slc5a1* expression is increased in the duodenal of individuals with impaired glucose tolerance and T2DM (30). *Slc5a1* activity remains unknown in the liver. We found that *Slc5a1* expression was induced in the liver of DIO mice by MET, suggesting that glucose uptake by liver may be increased by MET in the improvement of insulin sensitivity. *Slc5a1* expression was not changed in the colon of DIO mice and MET-treated DIO mice.

In this study, mRNA expression of *Slc5a9* and *Slc25a24* was elevated in the liver of DIO mice and the expression was further increased by MET. *Slc5A9* is also known as SGLT4, a sodium-dependent glucose transporter, which acts as an essential transporter for mannose, fructose and 1,5-anhydro-D-glucitol in the intestine and kidney (31). A mutation within *Slc5a9* gene is reported in the development of proliferative diabetic retinopathy (32). However, *Slc5a9* activity remains unknown in the liver. We observed that *Slc5a9* expression was induced by MET, suggesting that MET may promote uptake of mannose, fructose and 1,5-anhydro-D-glucitol in the liver through induction of *Slc5a9*. In DIO mice, it was reported that *Slc25a24* expression was increased in the white adipose tissues (WAT) of DIO mice during tissue expansion, and *Slc25a24*-knockout mice exhibited an obesity-resistant phenotype (33). This study suggests that *Slc25a24* may be required for adipose tissue expansion and a novel candidate gene in the control of obesity.

We found that *Slc6a14* and *Slc16a5* were upregulated by MET in the DIO mice, which is consistent with the published studies. *Slc6a14* is a Na^+^ and Cl^−^ dependent transporter for glutamine and other amino acids except glutamate and aspartate (34). Sathish Sivaprakasam, et al. suggested that the deficiency of *Slc6a14* was related to obesity, and dietary/pharmacologic interventions*-*induced *Slc6a14* expression in the intestinal tract might play a role in the prevention of obesity (35). *Slc16a5* is known as monocarboxylate transporter 6 (MCT6), which transports bumetanide in a pH- and membrane potential-sensitive manner independently of proton gradient (36). Lu, et al. reported that the treatment of mice with fenofibrate resulted in 3- to 6-fold upregulation of *Slc16a5* in the liver of mice, which was associated with an improvement in insulin sensitivity (37). After 24-hour fasting, *Slc16a5* was also detected as the 6th most increased gene with about a 5-fold upregulation in gene expression, compared with gene expression after a normal diet (38). These studies confirm that *Slc6a14* and *Slc16a5* may play a role in lipid metabolism due to its differential regulation under various interventions. Our data suggest that MET may increase liver uptake of amino acids and bumetanide in the improvement of insulin sensitivity and obesity.

In the liver analysis by RNA-seq, there are three other studies in the literature. Those were designed to understand MET mechanism in the regulation of liver gene expression in the normal mice or human hepatocytes. These studies provide evidence for MET direct impact in gene transcription. Meng Y et al. revealed that metformin changed gene expression profile in the mouse liver, which was associated with the beneficial/deleterious effects in the healthy mice (39). Lien F et al. found that MET regulated the bile acid homeostasis through gene transcription mediated by the AMPK-FXR pathway in the liver of normal mice (40). Luizon MR et al. reported that the AMPK-ATF3 pathway was activated by MET to regulate gene transcription in the normal human hepatocytes in the cell culture, which was associated with inhibition of gluconeogenesis by MET (41). We analyzed the RNA-seq data in the study by Luizon, et al, to understand the MET direct and indirect effect in the expression of *Slc* genes. The result suggests that MET does not alter expression of the 15 *Slc* genes identified in the DIO mice. The negative result from the cellular model suggest that MET may use an indirect mechanism in the restoration of the 15 *Slc* genes in the liver of DIO mice in current study. The restoration may a secondary effect of improved insulin sensitivity or hepatocyte function in the liver of DIO mice.

Although the mechanism by which MET restored the 15 *Slc* gene expression remains unclear, significance of *Slc* genes in the pathogenesis of metabolic disease is supported by a couple of studies. A study suggests that *Slc*13a5 expression is required for development of diet- and aging-induced obesity. *Slc*13a5 gene inactivation were protected the knockout mice from high-fat diet (HFD)- and aging-induced obesity, hepatic steatosis, and insulin resistance (42). Consistently, knockdown of human *Slc*13a5 resulted in lower lipid levels in a human hepatocyte cell line (43). *Slc*25a24 seems to have the same activity in the knockout mice, which were protected from HFD-induced obesity and hepatic steatosis (33). Conversely, a reduction in *Slc*16a11 activity promoted T2DM and induction of the gene expression decreased the risk of T2DM (44, 45). Similarly, a reduction in *Slc*30a8 activity increased the risk of T2DM, and overexpression of *Slc*30a8 improved glucose tolerance in mice (46, 47). These studies were conducted in the global transgenic mice, and the results might reflect the *Slc* activities in organs other than the liver.

In summary, we observed that more *Slc* genes exhibited alteration in the liver over the colon under in the DIO mice. In the liver of DIO mice, there were 34 *Slc* genes with mRNA alteration, in which 15 *Slc* genes were downregulated in mRNA expression, and the reduced expressions were restored in the liver by MET. The restoration of the *Slc* gene expression may be a secondary effect of the improved insulin sensitivity in liver to promote uptake of glucose, amino acids, mannose, fructose, 1,5-anhydro-D-glucitol and bumetanide in the hepatocytes. The *Slc* genes may contribute to liver metabolism through regulation of substrate exchange in hepatocytes. However, the possibilities remain to be tested in experiments. The upstream events for the *Slc* gene restoration remain to be identified in the MET action.

## Data Availability Statement

The datasets presented in this study can be found in online repositories. The names of the repository/repositories and accession number(s) can be found below: https://www.ncbi.nlm.nih.gov/, PRJNA735274.

## Ethics Statement

The animal study was reviewed and approved by The Institutional Animal Care and Use Committee (IACUC) of Shanghai Jiaotong University.

## Author Contributions

JY contributed to the concept and design of the study. JLe, YF, QH, XW, HJ, and XZ performed the experiments. JLe, YC, QW, PP, JLi, XL, and YZ processed the experimental data and prepared table and picture. JLe wrote the manuscript. JLe and JY revised the manuscript. JLe and JY are the guarantors of this work and, as such, had full access to all the data in the study and take responsibility for the integrity of the data and the accuracy of the data analysis. All authors contributed to the article and approved the submitted version.

## Funding

The study was supported by National Natural Science Foundation of China (81903961 to JLe) and the Natural Science Foundation of Shanghai (19ZR1424000) to JLe, and Construction project of Shanghai Key Laboratory of Molecular Imaging (18DZ2260400).

## Conflict of Interest

The authors declare that the research was conducted in the absence of any commercial or financial relationships that could be construed as a potential conflict of interest.

## Publisher’s Note

All claims expressed in this article are solely those of the authors and do not necessarily represent those of their affiliated organizations, or those of the publisher, the editors and the reviewers. Any product that may be evaluated in this article, or claim that may be made by its manufacturer, is not guaranteed or endorsed by the publisher.
