# Supplementary material for: Restoration of mRNA Expression of Solute Carrier Proteins in Liver of Diet-Induced Obese Mice by Metformin
Source: Front Endocrinol (Lausanne). 2021 Sep 30;12:720784. doi: 10.3389/fendo.2021.720784 (PMC8515182; doi:10.3389/fendo.2021.720784)
Supplement: Supplementary file 1 [file Table_1.docx]

Supplementary Table 1. qRT-PCR primer sequence of MET-responsive Slc transporters DEGs.

| Primer name | Forward primer (5'to3') | | Reverse primer (5'to3') |
| --- | --- | --- | --- |
| Slc2a10 | AAGAGCAGGACTCCAACGTG | CTTCTGCCGAACCCTGGAAA | |
| Slc2a13 | ACTACAGCGCAACCATCCTC | AAGGTAAGCTTCCTGCGACC | |
| Slc5a1 | CCAGTGGGCTGTACCAACAT | GAGAGTACTGGCGCTGTTGA | |
| Slc5a8 | CTGCCGAGGTCTACCGTTTT | AGGATTGTGCCACAGAGACG | |
| Slc5a9 | GGTTTTGACTAGGCCAGGCT | CTGGTTCCATTGCCACAAGC | |
| Slc6a14 | CAAAGTGTATTGCCGTGGGC | ATCTCTCCTGCCCCTATGCT | |
| Slc7a9 | GTACCACCATCACCTCTCTCC | TGATTTGCTGGGACAAACCAG | |
| Slc9a2 | GTCTGCTTTACCCTGGCCTT | GAAGGTTAGCGGGATGGTCC | |
| Slc9a3 | CCTGACCATTAAGCCCCTGG | GCTGAGGAACTTCCGGTCAA | |
| Slc13a2 | TCGCTCCTACCTGATTGTGC | AATGAGTGGCAGAGGCAACA | |
| Slc15a2 | GGTTTGCTATCTGCAACCGC | CAGAACATGGGCAGTGGGAT | |
| Slc16a5 | TGTGTGGCGTGACTTCTCTC | GCTTGCCCATCTTGCTTCAG | |
| Slc25a24 | GCTCCTTTGGATCGCCTCAA | TACCATCTGCCGAAAGCCTC | |
| Slc26a2 | GCCCCAGTCCATTGCTTACT | GCGGGATGTACCAAACAGGA | |
| Slc26a3 | GGCGGCAAGTGTAGCATTTC | TCAGGCTCATACACTTCGGC | |
| Slc34a2 | GCCCAGAACAAGAGCCTGAT | TTATCAGTCGAGGGGACGGT | |
| Slc37a1 | GTTTGTCTCCCTCTGCTGTGT | GCCACTCTAGGAGCCCTTAC | |
| Slc44a4 | TCGTCCTCCTGGTCATTTGC | TGTTGGACGCCCAGTAGATG | |
| Slc51b | GAGAAACCAGACTTGGCCCC | TCTCAACGCTCCCTAGCTCT | |
| Slc52a3 | TCGAGTTGCCCGAGGATCTA | GCCAAAGACGCAGACCAGTA | |
| GAPDH | GAACGGGAAGCTCACTGG | GCCTGCTTCACCACCTTCT | |
