# Supplementary material for: Restoration of mRNA Expression of Solute Carrier Proteins in Liver of Diet-Induced Obese Mice by Metformin
Source: Front Endocrinol (Lausanne). 2021 Sep 30;12:720784. doi: 10.3389/fendo.2021.720784 (PMC8515182; doi:10.3389/fendo.2021.720784)
Supplement: Supplementary file 2 [file Table_2.docx]

Supplementary Table 2. Slc transporters DEGs in colon (\log_2_FC\≥1 and P<0.01).

| **Comparison** | **Gene ID** | **Symbol** | **Description** | **log_2_FC** | **P value** | **Variation   trend** |
| --- | --- | --- | --- | --- | --- | --- |
| HFD VS NCD | 54403 | Slc4a4 | electrogenic sodium bicarbonate cotransporter 1 isoform a; electrogenic sodium bicarbonate cotransporter 1 isoform c; electrogenic sodium bicarbonate cotransporter 1 isoform b | -1.26344 | 0.003022 | Down |
|  | 269356 | Slc4a11 | sodium bicarbonate transporter-like protein 11 | 1.232523 | 0.00878 | Up |
|  | 64454 | Slc5a4b | solute carrier family 5 member 4b | -1.39669 | 0.006712 | Down |
|  | 74338 | Slc6a19 | sodium-dependent neutral amino acid transporter B (0) AT1 | -1.71627 | 0.001099 | Down |
|  | 105243 | Slc9a3 | sodium/hydrogen exchanger 3 precursor | -1.4882 | 0.004609 | Down |
| HFD+MET  VS HFD | 102680 | Slc6a20a | sodium- and chloride-dependent transporter XTRP3A | -1.16859 | 7.03E-05 | Down |
|  | 56857 | Slc37a2 | solute carrier family 37 (glycerol-3-phosphate transporter), member 2, isoform CRA_a, partial | -1.50486 | 0.001073 | Down |
