# Supplementary material for: Restoration of mRNA Expression of Solute Carrier Proteins in Liver of Diet-Induced Obese Mice by Metformin
Source: Front Endocrinol (Lausanne). 2021 Sep 30;12:720784. doi: 10.3389/fendo.2021.720784 (PMC8515182; doi:10.3389/fendo.2021.720784)
Supplement: Supplementary file 3 [file Table_3.docx]

Supplementary Table 3. The localizations, physiological functions and associated diseases of MET-responsive Slc transporters DEGs.

| **Gene symbol** | **Protein names** | **Localizations** | **Physiological functions** | **Associated diseases** |
| --- | --- | --- | --- | --- |
| Slc2a10 | ATS; ATORS; GLUT10; Slc2a10 | heart; lung; brain; liver; skeletal muscle; pancreas; placenta; kidney; adipose [1] | glucose transporter (GLUT10) [2] | arterial tortuosity syndrome (ATS); type 2 diabetes [2; 3] |
| Slc2a13 | HMIT; Slc2a13 | brain; adipose; kidney [4] | proton (H^+^) myo-inositol cotransporter (HMIT) [5] | Alzheimer’s disease [6] |
| Slc5a1 | NAGT; SGLT1; D22S675; Slc5a1 | heart; liver; skeletal; muscle; lung; trachea; gall bladder; colon; rectum; brain; blood vessels; uterus; breast; testis; pancreatic α cells [7; 8] | Na^+^- D-glucose cotransporter (NAGT, SGLT), glycemic control, intestinal glucose absorption and glucose-dependent incretin secretion [8; 9] | glucose-galactose malabsorption, diabetes and cancer [8] |
| Slc5a8 | AIT; SMCT; SMCT1; Slc5a8 | gastrointestinal tract; kidney; thyroid; brain; retina [10] | sodium-coupled  monocarboxylate transporter (SMCT), including butyrate, propionate, lactate and pyruvate [11]; apical iodide transporter (AIT) [12] | cancers, such as colon and breast cancer [10] |
| Slc5a9 | SGL; SGLT4; AI159731; Slc5a9 | liver; small intestine; kidney [13] | sodium-dependent glucose transporter (SGL, SGLT) for mannose, 1, 5-anhydro-D-glucitol, and fructose [13] | proliferative diabetic retinopathy [14] |
| Slc6a14 | BMIQ11; Slc6a14 | colon; lung; eye; mammary gland [15] | sodium/chloride dependent broad amino acid transporter ATB^0,+^ [15] | obesity [16]; cystic fibrosis [17]; male infertility [18] |
| Slc7a9 | BAT1; CSNU3; Slc7a9 | kidney; liver; small intestine; placenta [19] | sodium-independent transport of cystine and dibasic amino acids, b^o, +^ amino-acid transporter (BAT) [20] | non-type I cystinuria [20] |
| Slc9a2 | NHE2; Slc9a2 | intestine; kidney; stomach; ileal villus [21] | Na^+^ absorption with SCFA^−^ [22]; Na^+^/ H^+^ exchanger (NHE2) [21] | renal disease [23] |
| Slc9a3 | NHE3; DIAR8; NHE-3; Slc9a3 | jejunum; ileum; colon; stomach; liver; pancreas; gall bladder [24] | Na^+^/ H^+^ exchanger 3 (NHE3) [24] | congenital secretory sodium diarrhea [24] |
| Slc13a2 | NaCT; NADC1; SDCT1; NaDC-1;  Slc13a2 | kidney; intestine; brush border membrane [25] | Na^+^-dicarboxylate cotransporter 1 (NaDC1, SDCT1); succinate/citrate cotransporter [25] | kidney stone disease [25; 26] |
| Slc15a2 | PEPT2; Slc15a2 | kidney; choroid plexus; neurons; astrocytes (neonates); lung; mammary gland; spleen; enteric nervous system [27] | Di/tripeptides proton, proton-coupled oligopeptide transporter (POT) [28] | lead exposure (2 haplotype associated with higher blood lead burden in male children) [29] |
| Slc16a5 | MCT6; Slc16a5 | kidney; muscle; brain; heart; pancreas; prostate; lung; placenta [30] | human monocarboxylate transporter 6 (MCT6), transport of bumetanide [31] | disorders of glucose and lipid metabolism [32] |
| Slc25a24 | APC1; SCAMC1; SCAMC-1; Slc25a24 | testis; small intestine; heart; skeletal muscle; liver; pancreas [33] | mitochondrial inner membrane ATP-Mg/Pi carrier (APC1); short Ca^2+^-binding mitochondrial carriers (SCAMC1) [33] | craniosynostosis syndrome [34]; human progeroid syndromes [35] |
| Slc26a2 | DTD; EDM4; DTDST; MST153; D5S1708; MSTP157; Slc26a2 | colon; cartilage; sweat glands; bronchial glands; placental villi [36] | diastrophic dysplasia sulfate transporter (DTD, DTDST), SO_4_^2−^, Cl^−^, oxalate, or SO_4_^2−^/2 Cl^−^ exchanger [37] | chondrodysplasias, such as diastrophic dysplasia, atelosteogenesis type II, achondrogenesis type IB and multiple epiphyseal dysplasia [38] |
| Slc26a3 | CLD; DRA; Slc26a3 | lleum; colon; duodenum; pancreatic ducts; sweat glands; teeth; reproductive tract; gallbladder; stomach [39] | SO_4_^2−^/ Cl^−^or Cl^−^/ HCO_3_^−^ exchanger [40; 41]; uptake of SCFA^−^ [22] | down regulated in Adenoma (DRA) [42] ; congenital chloride diarrhea (CLD) [43] |
| Slc34a2 | PULAM; NaPi- IIb; NPTIIb; Slc34a2 | small intestine; colon; liver; lung; kidney; testis [44] | sodium-phosphate transporter (NaPi- IIb, NPTIIb) [45] | pulmonary alveolar microlithiasis (PULAM) [46] |
| Slc37a1 | G3PP; Slc37a1 | adult kidney, spleen, liver, small intestine, bone marrow; fetal liver, brain, spleen [47] | glycerol-3-phosphate (G3P) /Pi cotransporter (G3PP) [47] | colorectal cancer [48] |
| Slc44a4 | CTL4; NG22; TPPT; DFNA7; hTPPT1; C6orf29; Slc44a4 | colon; kidney; lung; testis; spleen; brain [49] | human thiamine pyrophosphate transporter (hTPPT) [50]; choline transporter-like protein 4 (CTL4) [51] | sialidosis, a lysosomal storage disease [52] |
| Slc51b | OSTB; OSTBETA; Slc51b | small intestine; liver; colon; kidney; testes; ovary; adrenal gland; mammary gland; uterus; prostate; thyroid; brain [53] | organic solute transporter (OSTβ), bile acid transporter, uptake of taurocholic acid [54] | congenital diarrhea and cholestasis [55] |
| Slc52a3 | RFT2; BVVLS; RFVT3; hRFT2; BVVLS1; C20orf54; bA371L19.1; Slc52a3 | testis; small intestine; prostate; placenta; colon; heart; kidney; brain; liver [56] | riboflavin transporter (RFT2, RFVT3) [56] | Brown-Vialetto-Van Laere syndrome (BVVLS) and Fazio-Londe disease [57] |

**REFERENCES**

[1] R. Augustin, The protein family of glucose transport facilitators: It's not only about glucose after all. IUBMB Life 62 (2010) 315-333.

[2] P.A. Dawson, J.C. Mychaleckyj, S.C. Fossey, S.J. Mihic, A.L. Craddock, and D.W. Bowden, Sequence and functional analysis of GLUT10: a glucose transporter in the Type 2 diabetes-linked region of chromosome 20q12-13.1. Mol Genet Metab 74 (2001) 186-199.

[3] F. Segade, Glucose transporter 10 and arterial tortuosity syndrome: the vitamin C connection. FEBS Lett 584 (2010) 2990-2994.

[4] M. Uldry, and B. Thorens, The SLC2 family of facilitated hexose and polyol transporters. Pflugers Arch 447 (2004) 480-489.

[5] M. Uldry, M. Ibberson, J.D. Horisberger, J.Y. Chatton, B.M. Riederer, and B. Thorens, Identification of a mammalian H(+)-myo-inositol symporter expressed predominantly in the brain. EMBO J 20 (2001) 4467-4477.

[6] Y. Teranishi, M. Inoue, N.G. Yamamoto, T. Kihara, B. Wiehager, T. Ishikawa, B. Winblad, S. Schedin-Weiss, S. Frykman, and L.O. Tjernberg, Proton myo-inositol cotransporter is a novel γ-secretase associated protein that regulates Aβ production without affecting Notch cleavage. FEBS J 282 (2015) 3438-3451.

[7] W.S. Lee, Y. Kanai, R.G. Wells, and M.A. Hediger, The high affinity Na+/glucose cotransporter. Re-evaluation of function and distribution of expression. J Biol Chem 269 (1994) 12032-12039.

[8] H. Koepsell, The Na-D-glucose cotransporters SGLT1 and SGLT2 are targets for the treatment of diabetes and cancer. Pharmacol Ther 170 (2017) 148-165.

[9] V. Gorboulev, A. Schürmann, V. Vallon, H. Kipp, A. Jaschke, D. Klessen, A. Friedrich, S. Scherneck, T. Rieg, R. Cunard, M. Veyhl-Wichmann, A. Srinivasan, D. Balen, D. Breljak, R. Rexhepaj, H.E. Parker, F.M. Gribble, F. Reimann, F. Lang, S. Wiese, I. Sabolic, M. Sendtner, and H. Koepsell, Na(+)-D-glucose cotransporter SGLT1 is pivotal for intestinal glucose absorption and glucose-dependent incretin secretion. Diabetes 61 (2012) 187-196.

[10] V. Ganapathy, M. Thangaraju, E. Gopal, P.M. Martin, S. Itagaki, S. Miyauchi, and P.D. Prasad, Sodium-coupled monocarboxylate transporters in normal tissues and in cancer. AAPS J 10 (2008) 193-199.

[11] S. Sivaprakasam, Y.D. Bhutia, S. Yang, and V. Ganapathy, Short-Chain Fatty Acid Transporters: Role in Colonic Homeostasis. Compr Physiol 8 (2017) 299-314.

[12] A.-M. Rodriguez, B. Perron, L. Lacroix, B. Caillou, G. Leblanc, M. Schlumberger, J.-M. Bidart, and T. Pourcher, Identification and characterization of a putative human iodide transporter located at the apical membrane of thyrocytes. J Clin Endocrinol Metab 87 (2002) 3500-3503.

[13] S. Tazawa, T. Yamato, H. Fujikura, M. Hiratochi, F. Itoh, M. Tomae, Y. Takemura, H. Maruyama, T. Sugiyama, A. Wakamatsu, T. Isogai, and M. Isaji, SLC5A9/SGLT4, a new Na+-dependent glucose transporter, is an essential transporter for mannose, 1,5-anhydro-D-glucitol, and fructose. Life Sci 76 (2005) 1039-1050.

[14] C. Ung, A.V. Sanchez, L. Shen, S. Davoudi, T. Ahmadi, D. Navarro-Gomez, C.J. Chen, H. Hancock, A. Penman, S. Hoadley, M. Consugar, C. Restrepo, V.A. Shah, J.F. Arboleda-Velasquez, L. Sobrin, X. Gai, and L.A. Kim, Whole exome sequencing identification of novel candidate genes in patients with proliferative diabetic retinopathy. Vision Res 139 (2017) 168-176.

[15] M. Scalise, L. Pochini, M. Galluccio, L. Console, and C. Indiveri, Glutamine transporters as pharmacological targets: From function to drug design. Asian J Pharm Sci 15 (2020) 207-219.

[16] E. Suviolahti, L.J. Oksanen, M. Ohman, R.M. Cantor, M. Ridderstrale, T. Tuomi, J. Kaprio, A. Rissanen, P. Mustajoki, P. Jousilahti, E. Vartiainen, K. Silander, R. Kilpikari, V. Salomaa, L. Groop, K. Kontula, L. Peltonen, and P. Pajukanta, The SLC6A14 gene shows evidence of association with obesity. J Clin Invest 112 (2003) 1762-1772.

[17] M. Ruffin, J. Mercier, C. Calmel, J. Mésinèle, J. Bigot, E.N. Sutanto, A. Kicic, H. Corvol, and L. Guillot, Update on SLC6A14 in lung and gastrointestinal physiology and physiopathology: focus on cystic fibrosis. Cell Mol Life Sci 77 (2020) 3311-3323.

[18] P. Noveski, M. Mircevska, T. Plaseski, B. Peterlin, and D. Plaseska-Karanfilska, Study of Three Single Nucleotide Polymorphisms in the SLC6A14 Gene in Association with Male Infertility. Balkan J Med Genet 17 (2014) 61-66.

[19] M. Palacín, The family of heteromultimeric amino acid transporters reveals aminoaciduria genes. Nephrol Dial Transplant 15 Suppl 6 (2000) 5-8.

[20] M. Palacín, G. Borsani, and G. Sebastio, The molecular bases of cystinuria and lysinuric protein intolerance. Curr Opin Genet Dev 11 (2001) 328-335.

[21] L. Fliegel, and O. Fröhlich, The Na+/H+ exchanger: an update on structure, regulation and cardiac physiology. Biochem J 296 ( Pt 2) (1993) 273-285.

[22] F. Stumpff, A look at the smelly side of physiology: transport of short chain fatty acids. Pflugers Arch 470 (2018) 571-598.

[23] M. Paillard, Na+/H+ exchanger subtypes in the renal tubule: function and regulation in physiology and disease. Exp Nephrol 5 (1997) 277-284.

[24] N.C. Zachos, M. Tse, and M. Donowitz, Molecular physiology of intestinal Na+/H+ exchange. Annu Rev Physiol 67 (2005) 411-443.

[25] M.J. Bergeron, B. Clémençon, M.A. Hediger, and D. Markovich, SLC13 family of Na⁺-coupled di- and tri-carboxylate/sulfate transporters. Mol Aspects Med 34 (2013) 299-312.

[26] K. Miyazawa, and K. Suzuki, [Gene expression and its role on urolithiasis]. Clin Calcium 21 (2011) 1473-1479.

[27] E. Viennois, A. Pujada, J. Zen, and D. Merlin, Function, Regulation, and Pathophysiological Relevance of the POT Superfamily, Specifically PepT1 in Inflammatory Bowel Disease. Compr Physiol 8 (2018) 731-760.

[28] H. Daniel, and G. Kottra, The proton oligopeptide cotransporter family SLC15 in physiology and pharmacology. Pflugers Arch 447 (2004) 610-618.

[29] D.E. Smith, B. Clémençon, and M.A. Hediger, Proton-coupled oligopeptide transporter family SLC15: physiological, pharmacological and pathological implications. Mol Aspects Med 34 (2013) 323-336.

[30] A.P. Halestrap, and D. Meredith, The SLC16 gene family-from monocarboxylate transporters (MCTs) to aromatic amino acid transporters and beyond. Pflugers Arch 447 (2004) 619-628.

[31] Y. Murakami, N. Kohyama, Y. Kobayashi, M. Ohbayashi, H. Ohtani, Y. Sawada, and T. Yamamoto, Functional characterization of human monocarboxylate transporter 6 (SLC16A5). Drug Metab Dispos 33 (2005) 1845-1851.

[32] R.S. Jones, C. Tu, M. Zhang, J. Qu, and M.E. Morris, Characterization and Proteomic-Transcriptomic Investigation of Monocarboxylate Transporter 6 Knockout Mice: Evidence of a Potential Role in Glucose and Lipid Metabolism. Mol Pharmacol 96 (2019) 364-376.

[33] G. Fiermonte, F. De Leonardis, S. Todisco, L. Palmieri, F.M. Lasorsa, and F. Palmieri, Identification of the mitochondrial ATP-Mg/Pi transporter. Bacterial expression, reconstitution, functional characterization, and tissue distribution. J Biol Chem 279 (2004) 30722-30730.

[34] N. Ehmke, L. Graul-Neumann, L. Smorag, R. Koenig, L. Segebrecht, P. Magoulas, F. Scaglia, E. Kilic, A.F. Hennig, N. Adolphs, N. Saha, B. Fauler, V.M. Kalscheuer, F. Hennig, J. Altmüller, C. Netzer, H. Thiele, P. Nürnberg, G. Yigit, M. Jäger, J. Hecht, U. Krüger, T. Mielke, P.M. Krawitz, D. Horn, M. Schuelke, S. Mundlos, C.A. Bacino, P.E. Bonnen, B. Wollnik, B. Fischer-Zirnsak, and U. Kornak, De Novo Mutations in SLC25A24 Cause a Craniosynostosis Syndrome with Hypertrichosis, Progeroid Appearance, and Mitochondrial Dysfunction. Am J Hum Genet 101 (2017) 833-843.

[35] K. Writzl, A. Maver, L. Kovačič, P. Martinez-Valero, L. Contreras, J. Satrustegui, M. Castori, L. Faivre, P. Lapunzina, A.B.P. van Kuilenburg, S. Radović, C. Thauvin-Robinet, B. Peterlin, A. Del Arco, and R.C. Hennekam, De Novo Mutations in SLC25A24 Cause a Disorder Characterized by Early Aging, Bone Dysplasia, Characteristic Face, and Early Demise. Am J Hum Genet 101 (2017) 844-855.

[36] S. Haila, J. Hästbacka, T. Böhling, M.L. Karjalainen-Lindsberg, J. Kere, and U. Saarialho-Kere, SLC26A2 (diastrophic dysplasia sulfate transporter) is expressed in developing and mature cartilage but also in other tissues and cell types. J Histochem Cytochem 49 (2001) 973-982.

[37] J.F. Heneghan, A. Akhavein, M.J. Salas, B.E. Shmukler, L.P. Karniski, D.H. Vandorpe, and S.L. Alper, Regulated transport of sulfate and oxalate by SLC26A2/DTDST. Am J Physiol Cell Physiol 298 (2010) C1363-C1375.

[38] P.A. Dawson, and D. Markovich, Pathogenetics of the human SLC26 transporters. Curr Med Chem 12 (2005) 385-396.

[39] U. Seidler, and K. Nikolovska, Slc26 Family of Anion Transporters in the Gastrointestinal Tract: Expression, Function, Regulation, and Role in Disease. Compr Physiol 9 (2019) 839-872.

[40] M.N. Chernova, L. Jiang, B.E. Shmukler, C.W. Schweinfest, P. Blanco, S.D. Freedman, A.K. Stewart, and S.L. Alper, Acute regulation of the SLC26A3 congenital chloride diarrhoea anion exchanger (DRA) expressed in Xenopus oocytes. J Physiol 549 (2003).

[41] N.M. Walker, J.E. Simpson, P.-F. Yen, R.K. Gill, E.V. Rigsby, J.M. Brazill, P.K. Dudeja, C.W. Schweinfest, and L.L. Clarke, Down-regulated in adenoma Cl/HCO3 exchanger couples with Na/H exchanger 3 for NaCl absorption in murine small intestine. Gastroenterology 135 (2008).

[42] C.W. Schweinfest, K.W. Henderson, S. Suster, N. Kondoh, and T.S. Papas, Identification of a colon mucosa gene that is down-regulated in colon adenomas and adenocarcinomas. Proc Natl Acad Sci U S A 90 (1993) 4166-4170.

[43] P. Höglund, S. Haila, J. Socha, L. Tomaszewski, U. Saarialho-Kere, M.L. Karjalainen-Lindsberg, K. Airola, C. Holmberg, A. de la Chapelle, and J. Kere, Mutations of the Down-regulated in adenoma (DRA) gene cause congenital chloride diarrhoea. Nat Genet 14 (1996) 316-319.

[44] H. Hilfiker, O. Hattenhauer, M. Traebert, I. Forster, H. Murer, and J. Biber, Characterization of a murine type II sodium-phosphate cotransporter expressed in mammalian small intestine. Proc Natl Acad Sci U S A 95 (1998) 14564-14569.

[45] H. Xu, L. Bai, J.F. Collins, and F.K. Ghishan, Molecular cloning, functional characterization, tissue distribution, and chromosomal localization of a human, small intestinal sodium-phosphate (Na+-Pi) transporter (SLC34A2). Genomics 62 (1999) 281-284.

[46] G. Castellana, G. Castellana, M. Gentile, R. Castellana, and O. Resta, Pulmonary alveolar microlithiasis: review of the 1022 cases reported worldwide. Eur Respir Rev 24 (2015) 607-620.

[47] L. Bartoloni, M. Wattenhofer, J. Kudoh, A. Berry, K. Shibuya, K. Kawasaki, J. Wang, S. Asakawa, I. Talior, B. Bonne-Tamir, C. Rossier, J. Michaud, E.R. McCabe, S. Minoshima, N. Shimizu, H.S. Scott, and S.E. Antonarakis, Cloning and characterization of a putative human glycerol 3-phosphate permease gene (SLC37A1 or G3PP) on 21q22.3: mutation analysis in two candidate phenotypes, DFNB10 and a glycerol kinase deficiency. Genomics 70 (2000) 190-200.

[48] D. Kikuchi, M. Saito, K. Saito, Y. Watanabe, Y. Matsumoto, Y. Kanke, H. Onozawa, S. Hayase, W. Sakamoto, T. Ishigame, T. Momma, S. Ohki, and S. Takenoshita, Upregulated solute carrier family 37 member 1 in colorectal cancer is associated with poor patient outcome and metastasis. Oncol Lett 15 (2018) 2065-2072.

[49] J.D. Ortigoza-Escobar, M. Molero-Luis, A. Arias, L. Martí-Sánchez, P. Rodriguez-Pombo, R. Artuch, and B. Pérez-Dueñas, Treatment of genetic defects of thiamine transport and metabolism. Expert Rev Neurother 16 (2016) 755-763.

[50] S.M. Nabokina, K. Inoue, V.S. Subramanian, J.E. Valle, H. Yuasa, and H.M. Said, Molecular identification and functional characterization of the human colonic thiamine pyrophosphate transporter. J Biol Chem 289 (2014) 4405-4416.

[51] E. Traiffort, S. O'Regan, and M. Ruat, The choline transporter-like family SLC44: properties and roles in human diseases. Mol Aspects Med 34 (2013) 646-654.

[52] J. Uhl, R. Penzel, C. Sergi, J. Kopitz, H.F. Otto, and M. Cantz, Identification of a CTL4/Neu1 fusion transcript in a sialidosis patient. FEBS Lett 521 (2002) 19-23.

[53] D.J. Seward, A.S. Koh, J.L. Boyer, and N. Ballatori, Functional complementation between a novel mammalian polygenic transport complex and an evolutionarily ancient organic solute transporter, OSTalpha-OSTbeta. J Biol Chem 278 (2003) 27473-27482.

[54] N. Ballatori, W.V. Christian, S.G. Wheeler, and C.L. Hammond, The heteromeric organic solute transporter, OSTα-OSTβ/SLC51: a transporter for steroid-derived molecules. Mol Aspects Med 34 (2013) 683-692.

[55] M. Sultan, A. Rao, O. Elpeleg, F.M. Vaz, B. Abu-Libdeh, S.J. Karpen, and P.A. Dawson, Organic solute transporter-β (SLC51B) deficiency in two brothers with congenital diarrhea and features of cholestasis. Hepatology 68 (2018) 590-598.

[56] Y. Yao, A. Yonezawa, H. Yoshimatsu, S. Masuda, T. Katsura, and K.-I. Inui, Identification and comparative functional characterization of a new human riboflavin transporter hRFT3 expressed in the brain. J Nutr 140 (2010) 1220-1226.

[57] A. Manole, P. Fratta, and H. Houlden, Recent advances in bulbar syndromes: genetic causes and disease mechanisms. Curr Opin Neurol 27 (2014) 506-514.
